# Non-invasive electrical stimulation restores corneal nerve density and function in diabetic neuropathy via KCNN-dependent mechanism

**DOI:** 10.21203/rs.3.rs-7895492/v1

**Published:** 2025-12-01

**Authors:** Menglu Yang

**Affiliations:** Schepens Eye Research Institute, Massachusetts Eye and Ear, Department of Ophthalmology, Harvard Medical School

**Keywords:** Transcutaneous electrical stimulation (ES), diabetes mellitus (DM), sensory function, neural regeneration, Ca2+-Induced K+ Channel (KCNN)

## Abstract

Diabetic neuropathy (DN) is the most common complication of diabetes mellitus (DM) and often involves the cornea, where progressive loss of nerve fibers contributes to impaired corneal sensitivity and wound healing defects. Current treatments are limited, underscoring the need for a regenerative therapy. Transcutaneous electrical stimulation (ES) is a neural modulating therapy that non-invasively delivers microcurrent electricity to the eye via orbital skin. ES treatment significantly restored the nerve density and sensory function in both streptozotocin-induced DM mice and *in vitro* isolated trigeminal ganglia (TG) neurons. Transcriptomics analysis of TGs from *in vivo* ES pointed to ion transport and Ca^2+^ signaling alteration. Consistently, membrane potential recording in TGs showed a rapid hyperpolarization upon ES accompanied by increased [Ca^2+^]_i_ level. Inhibition of Ca^2+^-Induced K^+^ Channel (KCNN) abolished the hyperpolarization and neural regeneration effect, whereas activation of KCNN channel significantly enhanced nerve regeneration in the STZ model compared with sham treatment. Overall, ES restores corneal nerve density and function in diabetes via KCNN activation, offering a novel, non-invasive, and clinically translatable therapeutic strategy for diabetic neuropathy.

## Introduction

Diabetes mellitus (DM) is a metabolic disorder that currently affects 537 million adults worldwide^1^, making it one of the most significant challenges to global health. Diabetic neuropathy (DN), characterized by distal-to-proximal loss of nerve function, is the most common complication, estimated to affect 50% of the DM patients^2^. DN presents as numbing or burning sensation, weakness of the limbs, and often leads to skin ulceration, infection, and eventually to amputation^3^, which creates a huge burden on patient health and healthcare systems. Among various affected tissues, the cornea is the most densely innervated in the human body and is particularly susceptible to DN^4^. Notably, it is also one of the most sensitive parts of the body, as the number of free epithelial nerve endings is estimated to be 300–600 times that of the skin^5^. The intrinsic transparency of the cornea allows detailed visualization of nerve fiber architecture, making the cornea a powerful model for studying peripheral neuropathies^6,7^. The corneal involvement of DN is characterized by progressive damage to corneal nerves and epithelial erosion^8^ - collectively referred to as diabetic keratopathy (DK). To date, therapeutic strategies to restore corneal innervation and function in DK remain poorly defined.

The majority of the corneal sensory nerve fibers originate in the trigeminal ganglia (TGs)^9^. It conducts touch, pain, and temperature, induces a blink reflex, stimulates wound healing^10^, provides trophic factors to the cornea, and induces tear and mucin production and secretion^11^. Progression of DK, which correlates with the severity of DN^8^, results in delayed wound healing, persistent epithelial damage, and corneal ulceration^12^ that harm vision. In addition, corneal nerve damage can lead to neuropathic corneal pain^12^, where patients suffer eye burning, stinging, and pain despite the loss of normal sensation. These conditions require prompt treatment to alleviate pain and restore vision. The current management of DN includes optimization of glucose and symptom management^3^. For cornea, recombinant human nerve growth factor offers a promising new therapeutic avenue^8^. However, the disadvantages of recombinant human nerve growth factor exist in the need for frequent applications (6 times per day), high cost, strict storage requirements, and eye discomfort^13^ and consequential intolerance. Creating more accessible and efficacious therapies is an urgent need for better nerve restoration and symptom relief to benefit a wider group of patients.

Transcutaneous electrical stimulation (TES) is an emerging neuromodulatory therapy that delivers micro-electrical currents non-invasively through the skin^14,15^. Electrical stimulation (ES) is progressively acknowledged as a clinical intervention for peripheral neuropathy^16,17,18^. However, the action of ES in DK has not yet been explored, nor did the mechanism of action behind the neuromodulation effect of ES. Herein, we demonstrate in a preclinical mouse model of DM, ES ameliorated corneal nerve loss and investigate the molecular events under ES in primary TG neuron culture.

## Result

### ES restores corneal nerve density and sensory function in STZ-induced DK models

**Figure 1:**
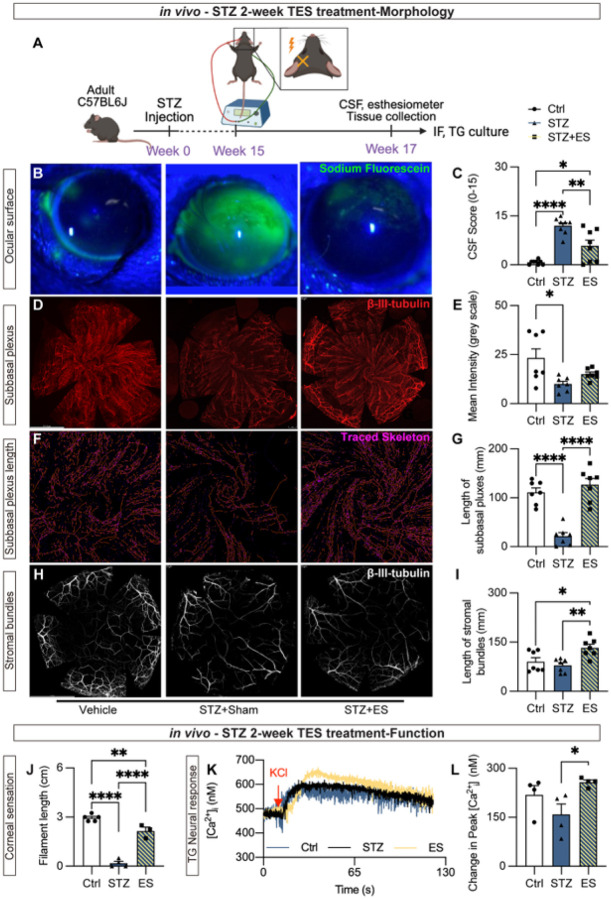
ES treatment in STZ: (A) Schematic of the experimental design. Yellow X indicates the site of the ES. (B) Corneal sodium fluorescein (CSF) staining of non-DM (Vehicle), STZ with sham treatment, and STZ with ES treatment. (C) CSF score using NIH scoring system. (D) β-III-tubulin staining in non-DM, STZ with sham treatment, and ES-treated STZ group. (E) Quantification of mean gray value from subbasal plexus signal of β-III-tubulin. (F, G) Subbasal plexus of the central cornea traced using ImageJ. (H, I) β-III-tubulin signal of the stromal bundle layer and quantification. n=7. (J) Coche-Bonet esthesiometer reading of the central cornea after 2-week ES treatment. (K) [Ca^2+^]_i_ over time recording of neurons isolated from STZ-induced T1DM mice with or without ES treatment, along with non-DM control. (L) Quantification of the peak of [Ca^2+^]_i_ change to baseline. n=4. * *p*< 0.05; ** *p*<0.01; ****, *p*<0.0001.

We induced type 1 DM (T1DM) in mice by STZ injection^19^, a well-established model that recapitulates the progressive metabolic and neuropathic features of human diabetes. In this model, corneal nerve loss was reported to start by 12 weeks post-STZ^20^. Mouse development of the T1DM status was validated by blood glucose ≥ 350mg/dl (**Supplemental Fig. 1**). Starting from week 15 post-injection, when DK has already developed, a random eye was treated with ES (ramp waveform, 300μA, 20Hz) for 4 min daily, the stimulation parameters identified previously as optimal for ocular surface protection^21^. This timing allowed us to directly test the therapeutic potential of ES in reversing corneal nerve pathology. Control mice received electrodes without electrical current (Sham). Mice received ES for 14 consecutive days, and the second day after the final ES, corneal fluorescein staining (CSF) was conducted to evaluate the epithelial integrity, while the corneal whole-mount were immunolabeled with a nerve-specific marker β-III-tubulin (Fig. 1A). At seventeen weeks post-STZ injection, the sham group demonstrated a significantly higher CSF score, indicating the loss of epithelial integrity, whereas this loss of epithelial integrity was mitigated by ES treatment (Fig. 1B and C). Consistently, in corneal wholemounts, the fluorescent intensity of β-III-tubulin labeling in the subbasal plexus was significantly reduced in STZ corneas compared to non-STZ controls, while ES treatment attenuated this reduction (Fig. 1D and E). Similarly, the total nerve length in the central cornea was significantly reduced in sham-treated STZ mice, which was again attenuated by ES treatment (Fig. 1F and G). Although STZ did not affect the total length of stromal nerve bundles, ES treatment resulted in a significant increase of stromal bundle length (Fig. 1I), indicative of a robust regenerative effect. These findings demonstrate that ES protects corneal epithelial integrity and promotes nerve regeneration, providing a structural basis for potential functional recovery.

We next investigated the functional benefits of ES on the corneal nerves. After 14 consecutive days of ES, the corneal sensation was evaluated in each group using a Cochet-Bonnet esthesiometer, which measures mechanical touch sensation (involving primarily mechanonociceptor excitability). At seventeen weeks post-STZ, the sham-treated group demonstrated a significant loss of sensory function, reflected by a shorter filament length needed to elicit a blink reflex; ES treatment for 14 days mitigated the sensory function reduction in STZ mice (Fig. 1J). Upon sacrifice, we isolated the trigeminal ganglia from the same animals and directly assessed neuronal function by measuring the intracellular [Ca^2+^] response to KCl, a widely used neuroexcitatory depolarizing stimulus (Fig. 1K). In the sham-treated group, the neuronal [Ca^2+^]_i_ response to K^+^ was reduced, although not statistically significant, likely due to the heterogeneity of TG neurons, which include multiple sensory subtypes (e.g., mechanical, thermal, and chemical) with variable excitability and susceptibility to diabetic injury. By contrast, 14 days of ES treatment significantly restored the neural [Ca^2+^]_i_ response (Fig. 1L). This result indicates that ES directly modulates the neural response to improve the mechanonociceptor function and improves epithelium integrity and corneal nerve density in STZ-induced preclinical model of diabetic mice.

### ES directly promotes axonal growth of trigeminal ganglia neurons

**Figure 2:**
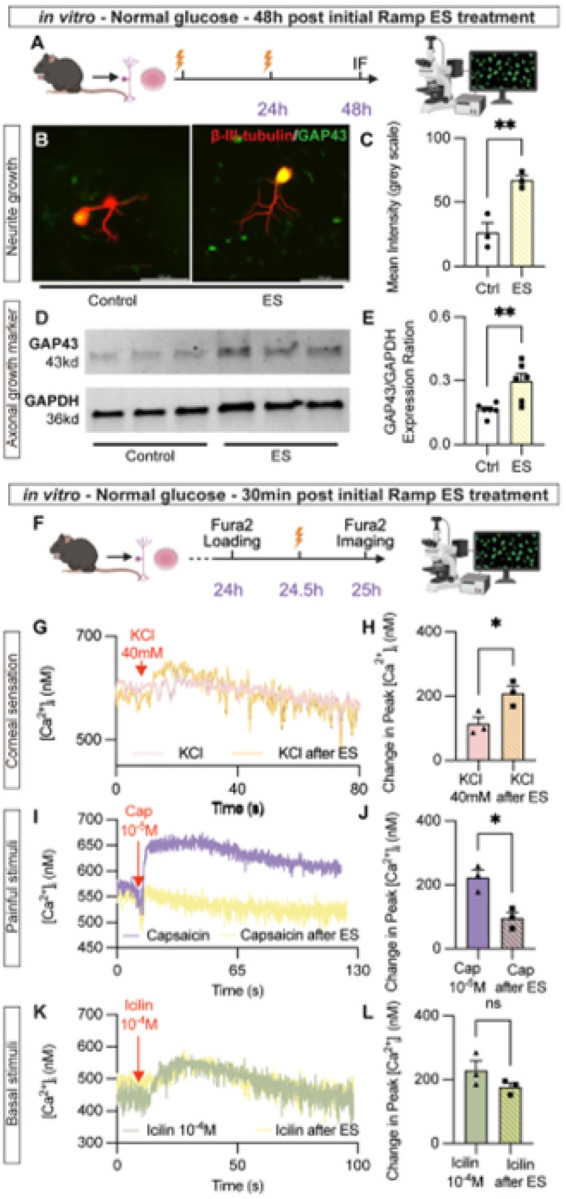
ES in isolated TG neurons: (A) Schematic of the experimental design. (B) IF signal of β-III-tubulin (red) and GAP43 (green) in primary TG neurons under sham treatment (left) and ES (right). (C) Quantification of the fluorescence intensity of GAP43. (D, E) GAP43 expression in primary TG culture is analyzed by WB and quantification. n=6. (F, G) Fura2 assay upon K^+^ 40mM stimulation, showing [Ca^2+^]_i_ over time and the change of peak [Ca^2+^]_i_ to baseline. (H, I) Fura2 assay upon TRPV1 agonist Capsaicin stimulation. (J, K) Fura2 assay upon TRPM8 agonist Icilin stimulation. n=3. *p< 0.05; ** *p*<0.01; ns, no significance.

We next determined whether ES directly acts on the corneal nerves to stimulate their growth or protect them against hyperglycemia stress using isolated TG neurons. In normal glucose conditions, ES (ramp waveform, 300μA, 20Hz) was applied for 30 min every 24h, and sham treatment group consisted of carbon electrodes without electrical current. This condition was optimized to prolong the ES effect during the limited in vitro culture period and to compensate for the direct neuronal exposure to electrodes in the absence of surrounding tissue^22^. Neurons were fixed at 48h after the first stimulation (Fig. 2A) and double-immunostained for antibodies against β-III-tubulin and growth-associated protein 43 (GAP43), an axonal growth marker (Fig. 2B). The results revealed significantly higher GAP43 levels in ES-treated neurons than sham-treated controls (Fig. 2C). Increased expression of GAP43 was also demonstrated by Western blot analysis under the same experimental setting (Fig. 2D and E), which further strengthens the finding that ES directly stimulates neurite outgrowth *in vitro*.

Next, we determined if ES protects the nerve fibers against a high glucose condition *in vitro*. TG neurons were isolated from naive C57BL6J mice and cultured in Neurobasal A media, supplemented with excessive D-glucose (100 mM) to mimic TIDM. Control cultures were supplemented with L-glucose (100 mM), which cannot be metabolized by cells, to serve as the osmolarity control. The neurons were cultured for 24 h after the treatment. β-III-tubulin immunolabeling (**Supplemental Fig. 3A**) revealed that high D-glucose significantly decreased the neurite length, as measured by the longest neurite, compared to L-glucose control; ES promoted longer neurite outgrowth under the high glucose condition (**Supplemental Fig. 3B**). Collectively, we show that ES directly acts on TG neurons to enhance axonal growth in both normal and high D-glucose conditions.

To evaluate the functionality of cultured trigeminal ganglia neurons, we conducted Fura2AM assay immediately after ES stimulation. Neuronal excitability was first evaluated using isotonic KCl as a general depolarizing stimulus. In ES-treated neurons, intracellular Ca^2+^ ([Ca^2+^]_i_) responses to KCl (40 mM) were significantly elevated compared to the sham-treated group (Fig. 2F and G), indicating that ES directly modulates the TG neuronal responsiveness that may underlie improved corneal sensory function. Because corneal innervation is critical not only for protective reflexes but also for ocular comfort and basal tear secretion, we next studied whether ES differentially affects pain-related nociceptive and thermo-sensory signals. Stimulation using the TRPV1 agonist capsaicin (10^− 5^M), which mimics painful stimulation (Fig. 2H) induced an increased [Ca^2+^]_i_ in sham-treated neurons. Remarkably, ES abolished the [Ca^2+^]_i_ response to capsaicin (Fig. 2I), suggesting that ES suppresses nociceptive activity and may alleviate ocular pain. In contrast, stimulation with TRPM8 agonist icilin with a final concentration of 10^− 4^M, which reproduces normal cold-sensation of the cornea and is essential for basal tear secretion, evoked similar [Ca^2+^]_i_ responses in both sham and ES-treated neurons (Fig. 2J and K). These results suggest that ES enhances overall TG neuron excitability, selectively dampens painful nociceptive signaling without impairing physiological cold sensing while promoting mechanonociceptor function. The data highlight a balanced modulation of TG neuronal subtypes underlying improved corneal recovery and ocular surface homeostasis.

### Transcriptomic profiling identifies Ca signaling and ion transport pathways mediated by ES

**Figure 3:**
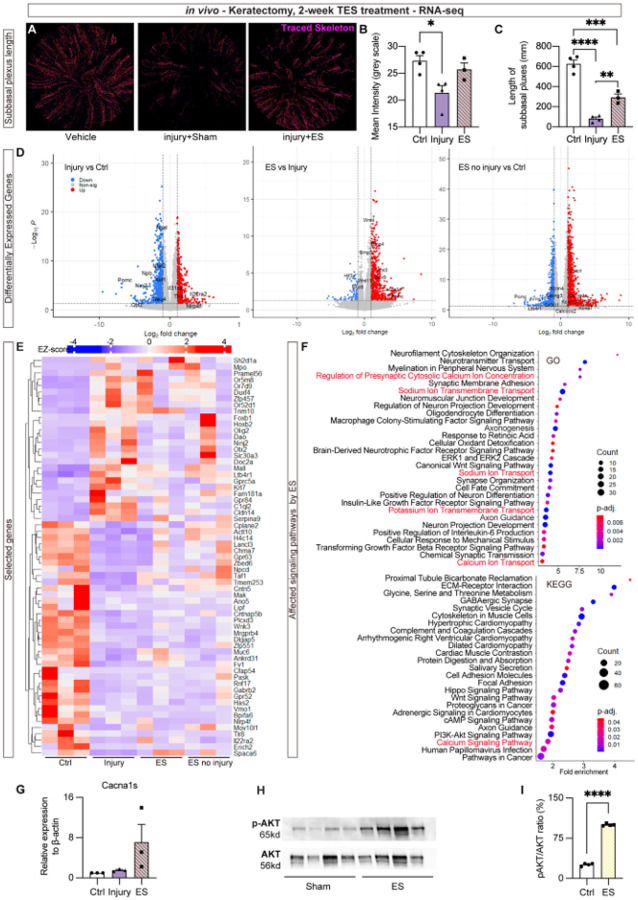
Transcriptomics analysis of 2-week-ES-treated TGs from keratectomy model: (A) β-III-tubulin signal traced at the wounded site in non-injured control, keratectomy injury with sham and ES treatment. (B). Quantification of the mean gray scale of β-III-tubulin signal. (C) Length of the subbasal plexus within the wound site. n=4. (D) DEG compared between each group visualized by volcano plots. (E) Hierarchical clustering of DEGs from each sample visualized by heatmap. n=3. (F) Enrichment analysis of DGEs between Injury and ES-treated group using Gene Ontology (top) and KEGG (bottom) database. (G) qPCR validation of Cacna1s. (H and I) WB validation of p-AKT and AKT in primary TG neuron treated with *in vitro* ES, and quantification of p-AKT/AKT ratio. Raw western blot shown in Supplementary Fig 5. n=4. *p< 0.05; ** *p*<0.01; ****, *p*<0.0001.

To further determine if the neural regenerative potential of ES extends to other sensory nerve conditions, we employed an acute mechanical corneal nerve injury model - the keratectomy wound. In this model, the superficial corneal nerve plexus was mechanically scrapped off in the central 1.5 mm diameter area. ES was applied 4 min daily for 14 consecutive days and the cornea was collected for wholemount immunolabeling of β-III-tubulin. The fluorescent intensity and subbasal plexus length was quantified at the wounded area. Significantly increased fluorescent intensity and plexus length was found in ES-treated corneas than sham control, indicating that ES robustly promotes reinnervation of the cornea following acute and severe nerve injury (Fig. 3A, B and C). These findings support the regenerative potential of ES across distinct models of both chronic (diabetic) and acute corneal nerve injury.

To investigate the mechanisms underlying the regenerative effects of ES, we performed RNA sequencing on mRNA isolated from mouse TGs under four conditions: untreated (Ctrl), 2 weeks after keratectomy wound (Injury), ES-treated keratectomy for 2 weeks (ES), and ES without keratectomy wound (ES no injury). The acute keratectomy model provides a well-defined and reproducible nerve injury paradigm that allows clearer distinction of ES-induced regenerative responses, minimizing the systemic metabolic confounders present in the diabetic model. This model enabled us to specifically capture transcriptional programs associated with ES-mediated nerve repair. A total of 22,084 expressed genes were identified. Of these, 6,859 genes were differently expressed (*padj* < 0.05) between untreated control and keratectomy injuries, 1,388 genes between sham-treated and ES-treated injuries, and 8,147 genes between non-treated control and ES no Injury groups (Fig. 3D).

Differentially expressed genes (DEG) between the untreated control and injury groups were identified using an adjusted *p*-value (padj < 0.05). To highlight the most biologically meaningful changes and minimize noise from modest expression shifts, we further applied a stringent cutoff of absolute log_2_(Fold Change) > 2.5. This threshold allowed us to focus on genes with robust alterations that are most likely to drive functional changes in TG responses. These genes were visualized in a heatmap (Fig. 3E). Gene ontology (GO) enrichment analysis of DEG between ES and Injury group revealed upregulation of genes related to neural development, axonogenesis, axon guidance, and synaptic organization/function (Fig. 3F, **upper panel**). Notably, several Ca^2+^ ion transport and signaling pathways were observed (Fig. 3F, **red)**. These results suggest that ES promotes nerve growth in part by regulating membrane ion channels and activating Ca^2+^ signaling. KEGG pathway analysis further demonstrated that ES regulates genes involved in synaptic function, axonogenesis, Wnt and Ca^2+^ signaling, as well as PI3K signaling pathway (Fig. 3F, **lower panel**). To understand the interconnection among these pathways, we performed gene network analysis, which identified genes involving Ca^2+^ signaling, namely *Unc79*, *Cacna1s*, and *Casq1* being upstream of regeneration-associated genes such as *Sox* and *Bmps* (**Supplemental Fig. 4**). These results suggest that Ca^2+^ signaling plays an initial regulatory role in ES-induced neural regeneration.

To validate the RNA-seq results, neurons from TG were isolated using CD90.2 magnetic beads to minimize glial contaminations, and the resultant RNAs were subjected to qPCR. We noted that the gene encoding L-type voltage gated Ca^2+^ channel showed a trend of increased expression by ES treatment, although it did not reach statistical significance (Fig. 3G), consistent with the RNA-seq data. The results further suggest that ES may act directly through modulating Ca^2+^ channel activity, without altering their expression levels. As AKT activation is known to occur downstream of Ca^2+^ signaling, we next asked if the AKT activation is altered. The AKT activation was confirmed using primary TG neurons, in which *in vitro* stimulation with ES resulted in a significant increase in AKT phosphorylation 15 minutes post-stimulation, as determined by WB analysis (Fig. 3H and I), indicating an activation of AKT by ES in consistent with the KEGG enrichment findings.

### ES induces [Ca] responses and activates KCNN channels to hyperpolarize TG neurons

Given the RNA-seq results on ion transportation and Ca^2+^ signaling, we investigated whether ES directly modulates membrane potential and [Ca^2+^]_i_ changes in TG neurons. To directly assess the electrophysiological responses to ES, isolated TG neurons from naive mice were cultured in F12 media supplemented with 10% FBS overnight to ensure cytoactivity. Neurons loaded with voltage-sensitive dye FluoVolt dye were recorded at 20 frames per second using real-time fluorescence microscopy.

**Figure 4:**
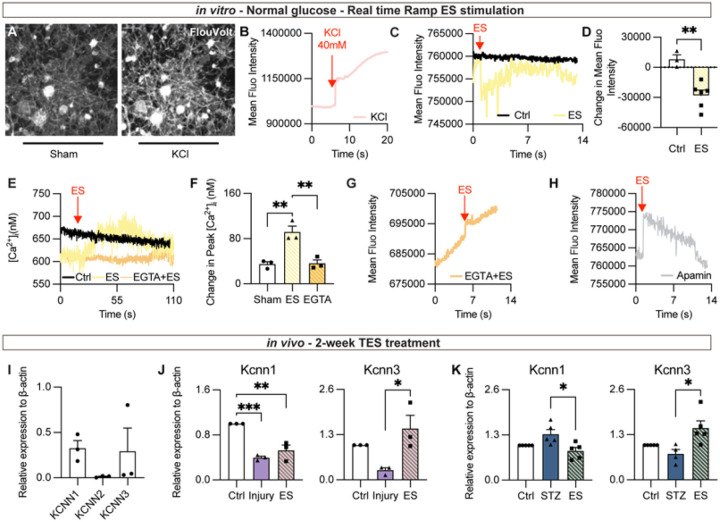
Membrane potential change of isolated TG neurons under continuous ES: (A) Representative image of TG neurons loaded with FluoVolt before and after KCl stimulation. Mean Fluorescence Intensity (MFI) over time is quantified in (B). MFI over time in neurons under ES (yellow) and control (black). The change in peak MFI to baseline is quantified in (D). n=5. (E) Fura 2 assay in isolated TG neurons under ES (yellow), with the presence of EGTA (orange), and control. Change in peak [Ca^2+^]_i_ to basal is quantified in (F). (G) MFI over time in neurons in response to ES with the presence of EGTA. (G) MFI over time in neurons in response to ES with the presence of KCNN inhibitor Apamin 10^−6^M. (I) Relative expression of Kcnn1-3 to β-actin in RNA extracted from naïve TGs. (J,K) Expression levels of *Kcnn* 1 and 3 in purified TG neurons in the keratectomy model (J) and STZ model (K) after 2-week-ES treatment. n=5. * *p*< 0.05; ** *p*<0.01. MFI, Mean Fluorescent Intensity.

Demonstrated in Fig. 4A, addition of KCl, a known stimulator of neural depolarization, significantly increased FluoVolt fluorescent intensity in TG neurons upon depolarization. Mean fluorescence intensity of each frame was quantified using ImageJ (Fig. 4B). Following 5–10s of baseline recording, ES (ramp, 100μA, 20Hz) was applied during the image acquisition. An immediate decrease of membrane potential was observed (Fig. 4C, **yellow**). The change in mean fluorescence intensity to baseline was quantified by ImageJ and plotted in Fig. 4D. This result shows that ES induces neuron hyperpolarization instead of depolarization.

Next, we recorded the [Ca^2+^]_i_ activity during ES using Fura2 assay. ES induced an immediate increase of [Ca^2+^]_i_ (Fig. 4E), and this ES-induced [Ca^2+^]_i_ surge was abolished by the Ca^2+^ chelator EGTA (**4E and F**). To further assess the relationship between Ca^2+^ influx and membrane potential, we blocked [Ca^2+^]_i_ response with EGTA. Strikingly, the ES-induced hyperpolarization visualized using FluoVolt signals was also blocked by EGTA pre-treatment (Fig. 4G), demonstrating that ES-induced hyperpolarization is [Ca^2+^]_i_-dependent. As potassium intermediate/small conductance calcium activated channel (KCNN) is known to be activated by [Ca^2+^]_i_ signal and mediate membrane hyperpolarization^23^, we tested whether it is involved in this process. Repeated the recording with KCNN blocker Apamin (10^− 6^M) pre-treatment showed that Apamin altered ES-induced hyperpolarization to depolarization (Fig. 4H). Hereto, we show that ES-induced hyperpolarization in TG neurons is Ca^2+−^dependent and mediates through KCNN channels.

There are three well-studied isotypes of KCNN channels, namely KCNN1, KCNN2, and KCNN3. Real-time qPCR from TG of naive mice detected expression of *Kcnn1* and *Kcnn3*, but not *Kcnn2* (Fig. 4I). To define the neuronal expression of *Kcnn1* and 3, total RNAs were extracted from TG neurons isolated using CD90.2-conjugated magnetic beads. Results of qPCR confirmed *Kcnn1* and *Kcnn3* expression in TG neurons. *Kcnn1* expression was significantly decreased in the injured group and stayed low regardless of ES treatment (Fig. 4J). In contrast, *Kcnn3* expression was significantly increased by ES compared to the sham-treated group (Fig. 4J). Similarly, in STZ-induced diabetic mice, *Kcnn1* expression was downregulated by ES treatment (Fig. 4K), while *Kcnn3* expression was significantly increased by ES compared to sham-treated STZ mice (Fig. 4K). These data indicate that ES selectively increases expression of *Kcnn3* in both injury and diabetic neuropathy models, suggesting that KCNN3 may play a key role in mediating the ES-induced neuronal hyperpolarization.

### ES-induced nerve regeneration is KCNN-dependent

**Figure 5:**
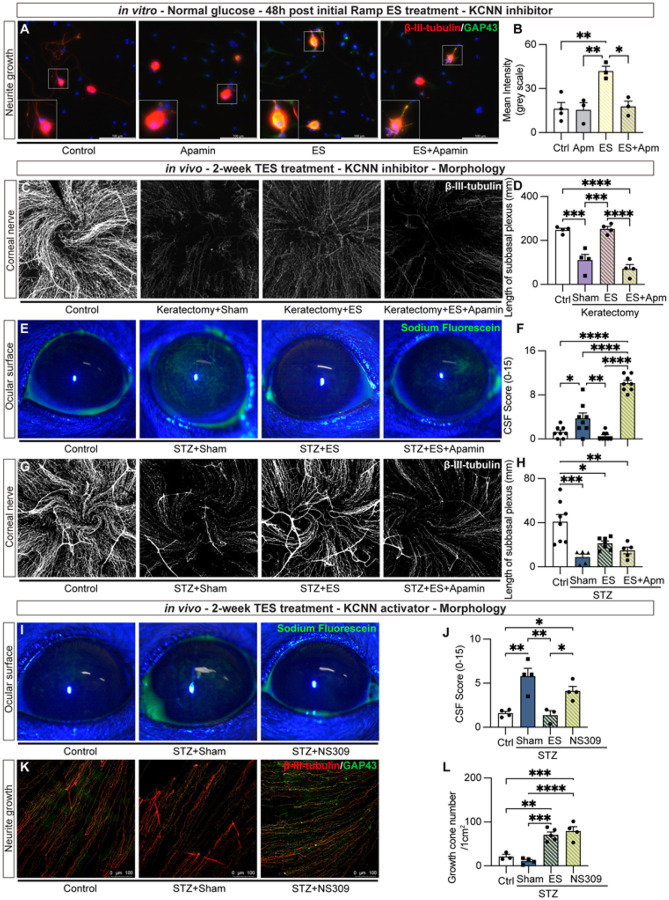
ES treatment with the presence of KCNN antagonist and agonist: (A) Representative images of IF labeling for β-III-tubulin (red) and GAP43 (green) in primary TG neurons under sham treatment, Apamin, *in vitro* ES, and ES combined with Apamin. (B) Quantification of the fluorescence intensity of GAP43. (C) Corneal wholemount with β-III-tubulin staining of the corneal nerve in non-injured control, keratectomy injury with sham treatment, ES-treated injury, and ES-treated injury with Apamin groups. (D) Quantification of the total length of central corneal plexus. (E, F) Corneal sodium fluorescein staining image of non-DM control, STZ with sham treatment, ES-treated STZ, and ES-treated STZ with Apamin, and quantification. (G, H) Corneal wholemount with β-III-tubulin immunolabeling in STZ model, and the total length of central corneal plexus was quantified. n=5 (I, J) Corneal images of sodium fluorescein staining taken from non-DM control, STZ with sham treatment, and KCNN agonist NS309-treated STZ groups, the NIH score is quantified. (K, L) The corneal wholemount with β-III-tubulin and GAP43 staining are shown, and the average numbers of growth cones are provided. n=4. *p< 0.05; ** *p*<0.01; ****, *p*<0.0001.

To investigate the role of KCNN in ES-induced nerve regeneration, we first investigated in the *in vitro* model. After neuron attachment post isolation, KCNN inhibitor Apamin (MCE, HY-P0256) was added to the Neuralbasal A media 10 min before ES (100μA, 20Hz, for 30 min). Neurons receiving ES without Apamin served as positive controls, while Apamin-treated neurons without ES as vehicle controls. Forty-eight hours later, IF with GAP43 and β-III-tubulin antibody showed that Apamin treatment alone did not affect GAP43 level in TG neurons. As expected, ES significantly increased GAP43 expression, whereas this effect was markedly diminished when ES was combined with Apamin treatment (Fig. 5A and B).

To model diabetic conditions *in vitro*, TG neurons were cultured in high glucose (100 mM) with L-glucose serving as osmolarity control. After neuron attachment post incubation, Apamin was added to high glucose-conditioned TG neurons 10 min prior to ES. The neurons were fixed and subjected to IF with β-III-tubulin antibody 24h later, and the length of the longest neurite was quantified. High glucose significantly reduced the neurite length compared to the osmolarity control, while ES application preserved the neurites. Apamin application abolished the effect of ES (**Supplemental Fig. 6**). These results indicate that the KCNN activation by ES contributes to axonal growth promotion and neural protection *in vitro*.

We next investigated the effect of KCNN blockage *in vivo* using the corneal keratectomy model. ES was applied for 14 consecutive days, with or without topical Apamin (10^− 6^M) treatment. Mice receiving electrodes without stimulation served as sham controls, and uninjured mice served as baseline controls. Consistent with our *in vitro* findings, ES significantly promoted the reinnervation of the central cornea, while this effect was significantly diminished by KCNN blockage with Apamin (Fig. 5C and D). Next, we evaluated the *in vivo* effect of KCNN in the STZ model. ES was applied for 14 consecutive days, with or without topical Apamin (10^− 6^M) treatment. Mice receiving electrodes without stimulation served as sham controls, and non-STZ mice served as baseline controls. On day fourteen of the ES treatment, corneal sensation was measured by a Cochet-Bonnet esthesiometer before tissue collection. The increased corneal sensation was observed in the ES treatment group compared to the sham-treated STZ group, and this effect was blocked by Apamin (**Supplemental Fig. 7**). Corneal fluorescein staining revealed significantly decreased epithelial disruption in the ES-treated STZ group compared to the sham-treated group, an effect which was also abolished by Apamin (Fig. 5E and F). Similar results were demonstrated using the total length of central subbasal plexus as a readout in the keratectomy model, where Apamin treatment reversed the ES-induced nerve regeneration in STZ model (Fig. 5G and H). Together, these results indicate that activation of KCNN is essential for ES-induced nerve regeneration and sensory recovery.

To determine if KCNN activation is sufficient for corneal nerve regrowth, starting from week 17 after STZ induction, KCNN agonist NS309 (10^− 4^M) was applied topically to both eyes of the STZ mice once daily for a consecutive 14 days. STZ mice receiving sham treatment with topical saline eye drops served as vehicle controls, while STZ mice receiving ES treatment served as positive controls. On day 14, the CSF was measured, and the corneas were collected for wholemount IF against β-III-tubulin and GAP43. The result showed that similar to ES treatment, NS309 significantly diminished the increased CSF score induced by STZ (Fig. 5I and J). GAP43 staining showed that both NS309 and ES treatment significantly increased the number of growth cones compared to sham-treated corneas (Fig. 5K and L). These results indicate that the activation of KCNN plays a critical role in driving the regeneration of corneal nerves in both diabetes and mechanical injuries.

## Discussion

In the current study, we demonstrated that ES successfully promoted corneal reinnervation in DK, the corneal manifestation of DN, restoring both structural innervation and functional sensation. It enhanced corneal sensory function, restored epithelial integrity, and increased subbasal nerve density. Mechanistically, ES induced nerve regeneration by triggering a rapid Ca^2+^ influx that drove membrane hyperpolarization through KCNN activation (**Fig. 7**). Importantly, blockade of Ca^2+^ influx or KCNN activity abrogated ES-induced hyperpolarization *in vitro* and nerve regeneration *in vivo*. Taken together, our findings indicate that ES promotes corneal nerve regeneration through Ca^2+^-KCNN signaling axis and contributes to overall corneal homeostasis by restoring innervation, improving sensation, and maintaining epithelial integrity. The data highlight the therapeutic potential of ES for neurodegenerative conditions such as DK.

Our findings extend previous work on ES as a regenerative therapy in both the peripheral and central nervous system. ES has attracted great interest in the recent decade in the motor nervous system^17^. It has been published that ES enhanced corneal reinnervation in mechanically wounded corneas without preexisting disease background^24,25,26^, our result demonstrates broader applications for targeting disease-associated corneal nerve degeneration. The *in vivo* relevance of these findings was confirmed in two distinct models: corneal keratectomy and STZ-induced diabetes. The mechanical wound model is widely used in corneal nerve regeneration research, as it provides a fast and controllable corneal nerve damage. STZ-induced diabetes model better mimics the corneal nerve damage caused by diabetic neuropathy, providing insights into the therapeutic role of ES in disease-induced nerve damage. The finding that ES promoted corneal nerve regeneration and epithelial integrity highlights the functional consequences of TG neuron modulation on the ocular surface. DK as a common and vision-threatening complication of diabetes with limited treatment options, the therapeutic implications of these findings are significant.

Our results provide important mechanistic insight into how ES regulates TG neuronal function. Calcium imaging and our *in vitro* assays revealed that ES induced a surge in intracellular Ca^2+^ and evoked membrane hyperpolarization through KCNN activation. Published works have shown that ES elevates intracellular Ca^2+^ level ([Ca^2+^]_i_)^27^, activates phosphoinositide 3-kinases (PI3Ks)^28,29^, and promotes Brain-Derived Neurotrophic Factor (BDNF) to promote axonal growth. Our results align with the published work with the observation of Ca^2+^ elevation, AKT activation, as well as increased BDNF receptor signaling pathway, as suggested in GO analysis. In contrast to inducing nerve depolarization, as reported by others^30^, our data showed that ES hyperpolarizes the TG neurons through an involvement of potassium channels. One potential explanation is that the hyperpolarization we observed is specific to the ramp waveform we used, while other groups commonly use rectangular waveforms. It has been reported that in alpha motor neurons, the gradually changing amplitude of the stimulating current, as in ramp waveforms, decreases the amplitude of neural depolarization^31^. Work in retinal ganglion cells indicates that neural activation depends on the amplitude and frequency of stimulation^32^, indicating the membrane potential changes in response to ES are highly waveform-dependent.

The hyperpolarization induced by ES was abolished by both Ca^2+^ chelation and the KCNN blocker Apamin. The KCNN is a family of K^+^ channels that is activated upon calmodulin binding regardless of the membrane potential^33^. During [Ca^2+^]_i_ surge, calmodulin captures Ca^2+^ and binds to KCNN to induce an outflow of K^+^ that leads to hyperpolarization. Our data suggest that KCNN directly regulates neuronal growth, which is likely due to the hyperpolarization state that has switched the neuron from a conductive to a regenerative state. Hyperpolarization has been well recognized in enhancing memory and learning activities^34,35^, and most recent evidence unveiled its role in promoting neurogenesis in central nerve system development^36^. By identifying KCNN-dependent Ca^2+^ signaling as a key pathway linking ES to neuronal survival and regeneration, this study provides new mechanistic insights into how ES promotes functional recovery in the nervous system.

An additional novel observation of this study is that ES differentially regulates TG sensory subtypes. Previous testing of corneal nerve function, which relies primarily on touch stimulation, which activates mechanonociceptors. As mentioned, corneal sensory nerves carry the sensations of touch, pain, and temperature, stimulate the blink reflex, and regulate tear production through nociceptors^10^, namely TRPV1 and TRPM8. TRPV1 expressed on the corneal nerves senses noxious stimuli and is the main contributor of neuropathic pain in DM^37,38^. TRPM8 is activated by innocuous cold temperatures^39^ and maintains basal tear secretion^40,41^. Notably, we observed ES inhibited the neuron response to a TRPV1 agonist capsaicin but did not alter the response to TRPM8 agonist icilin *in vitro*. This selective modulation indicates that ES not only promotes structural regeneration but also regulates a homeostatic corneal nerve function without exacerbating pain or disrupting normal thermosensation. Future studies employing more advanced models, such as iPSC-derived sensory neurons, will be critical to clarify the mechanistic insights involved and ultimately identify novel therapeutic targets that alleviate ocular pain without impairing the normal sensory functions essential for ocular homeostasis. Together, the demonstration that our ramp waveform ES can selectively suppress nociceptive activity while enhancing mechanosensory responses distinguishes this approach from nonspecific pro-regenerative strategies and suggests therapeutic specificity.

This study primarily focuses on the effect of ES on corneal nerve damage; therefore, we did not include the situation when ES is applied to DK eyes when DM is under treatment. The prolonged effect of ES on corneal nerves is also unclear. Our future scope is to address these limitations.

In conclusion, non-invasive ES treatment significantly promoted corneal nerve regeneration, restored epithelial integrity, enhanced neurite outgrowth in both DK and corneal injury models. ES selectively suppressed nociceptive signaling and preserved and/or promoted physiological cold- and touch-sensing, thereby balancing distinct sensory modalities for corneal homeostasis. These findings demonstrated the potential of ES as a non-invasive therapeutic approach for diabetic keratitis, neuropathy and other neurodegenerative conditions.

## Materials and Method

### Animals:

All C57BL6J mice were purchased from Jackson Lab (Bar Harbor, ME). We used a modified STZ-induced type I DM (T1DM) model on male 8-week-old C57BL/6J as we published^19^. STZ (Sigma, MO) 30mg/kg was mixed with saline and immediately injected intraperitoneal for a consecutive 5 days. Blood glucose levels were measured two weeks after the initial injection, and mice with glucose levels exceeding 350 mg/dL were considered diabetic. For mechanical injury, we used a keratectomy model on both male and female C57BL/6J mice at 8 weeks of age, following the published method^42^. Mice were anesthetized using a single intraperitoneal (IP) injection of ketamine and xylazine. Anesthetized mice were placed on heating pads to maintain body temperature while sedated and continue to be placed on a warm pad within their cage till recovery. Once sedation is confirmed, the central 1.5 mm diameter area was marked using a biopsy punch and the epithelium and the upper 1/3 of the corneal stroma of a random eye was removed using a rotating spur. All mice were kept in a 12-hour light/dark cycle with free access to food and water. All animal experiments were performed following protocols approved by the Institutional Animal Care and Use Committee of the Schepens Eye Research Institute and followed the Association for Research in Vision and Ophthalmology (ARVO) standards of using animals in research No. 2023N000038. The animal experiments adhered to the ARRIVE guidelines (https://arriveguidelines.org)^43^.

#### Primary TG neuron isolation, culture, and purification

C57BL/6J mice aged 8–12 weeks were used for TG neuron isolation and primary culture, following the method published by Malin, et al^44^. Fresh TG tissues were minced and enzymatically digested in a cocktail containing collagenase II and dispase II for 30 min at 37°C. The resulting cell suspension was purified by Percoll gradient centrifugation in L15 medium supplemented with 10% FBS. The cell pellet was resuspended in Neurobasal A medium and plated onto dishes precoated with laminin and poly-D-lysine. To purify neurons from glial cells, the cell pellet was resuspended in 90μl Neural Basal A media with 10μl CD90.2 magnetic beads (Miltenyi Biotec, Bergisch Gladbach, Germany), and incubated at room temperature for 20 min. The mixture was then loaded into MS Columns on a magnetic separator (Miltenyi Biotec, Bergisch Gladbach, Germany). The flow-through was discarded, and the purified neurons were pushed out in autoMACS rinsing solution (Miltenyi Biotec, Bergisch Gladbach, Germany) by a plunger. The isolated neurons were then pelleted by centrifuging at 400g for 6 min and proceeded for future assays.

#### Transcutaneous Electric Stimulation *in vivo*

*In vivo* ES was performed as previously described^21^ using the STG4000 pulse generator (Multichannel Systems, Reutlingen, Germany). Under isoflurane anesthesia, the anode electrode was positioned on the mouse’s abdomen through the conductive gel (Spectral 360; Parker Laboratories, Fairfield, NJ, USA). The cathode electrode probe was applied to the skin over the orbital area through a conductive gel interface. For sham controls, electrode probes were applied to the orbital area without delivering an electrical current. A biphasic ramp waveform (300 μA, 20 Hz) was administered for 4 minutes daily over 14 days. To minimize potential confounders, mice were subjected to ES in a randomized order.

#### Electric stimulation *in vitro*

Electric stimulation of cell cultures was conducted using the STG4000 pulse generator (Multichannel Systems, Reutlingen, Germany) with a biphasic ramp waveform (100 μA, 20 Hz, 30 min). The electrical current was applied to the cultures via a c-dish carbon electrode plate (Ion Optix, Westwood, MA, USA). To ensure sterility, the c-dish was incubated in 70% ethanol for 15 minutes, followed by a 15-minute rinse with distilled water, and subsequently air-dried for 1 hour before reuse^21^.

#### Corneal Fluorescein Staining (CSF)

One μL of 2.5% fluorescein (Sigma-Aldrich Corp., St. Louis, MO, USA) was applied to the lateral conjunctival sac, and staining scores were recorded after eye examination using slit-lamp microscopy (Topcon SL-DC4, Tokyo, Japan) under cobalt blue light. The punctate staining of the ocular surface was evaluated in a masked fashion and graded as per the National Eye Institute Scoring System (Bethesda, MD, USA), giving a score between 0 and 3 for each of the five areas of the cornea. Each cornea was scored 3 times individually by different investigators, and the average score of each time was recorded as the final score.

#### Corneal sensation measurement

A Cochet-Bonnet esthesiometer was used for measuring corneal sensation. Mice were acclimated to the testing environment by holding scruff of the neck for several seconds before testing, by an investigator masked to the experimental group. The filament was extended to 6 cm and a gentle touch was applied to the tip of the nylon filament to the central cornea. The filament length was decreased every 0.5 cm to repeat the touch if no blink response is observed, until a consistent blink response is induced. Each length was repeated for 6 times to ensure accuracy. The length of the filament was recorded as the sensory function of the cornea.

### Corneal whole mount and IF:

The corneal wholemount was conducted following the published method^45^. The eyes were immediately enucleated and fixed with 1.3% paraformaldehyde (VVR Life Science, Radnor, PA, USA) at room temperature for 1 h. The corneas were then dissected, and permeabilized in 1% Triton X-100 for 1 h at room temperature, followed by blocking and in 0.2% Triton X-100 1% BSA for 30 min. The samples were then incubated with a primary antibody to β-III-tubulin (1:30; R&D, NL1195R, Minneapolis, MN) for 24 h at 4°C then 2 h at room temperature and washed with PBS 3 times for 10 min. Corneas were mounted on slides under the microscope and covered with a ProLong Diamond antifade reagent (Thermo Fisher Scientific, Waltham, MA, USA). The cornea was then imaged using a Z scan with 4×4 tile scan by confocal microscopy (Leica SP8, Wetzlar, Germany). To quantify the nerve length, a central area of 400 pixel^2^ was selected and transformed into a binary image. The nerve was traced, and the total length is quantified by Skeleton 3D plugin using ImageJ 1.54p as described in^45^.

#### FluoVolt assay

Isolated TG neurons from naive C57BL6J mice were cultured in F12 media with 10% fetal bovine serum (FBS) overnight to ensure cytoactivity during living cell analysis. Neurons were loaded with FluoVolt dye (ThermoFisher, MA, USA) in KRB buffer for 30min. ES was applied to the neurons during the image acquisition and the FlouVolt signal was visualized using time-lapse microscopy (Leica Dmi8, Wetzlar, Germany) at 20 frame/s. The mean fluorescence intensity was quantified by ImageJ.

#### [Ca^2+^]_i_ measurement

Primary TG neurons were plated onto 35-mm glass-bottom culture dishes and incubated at 37°C overnight as described previously^21^. Cells were then incubated for 1 h at 37°C with Krebs-Ringer bicarbonate buffer containing 119 mM NaCl, 4.8 mM KCl, 1.0 mM CaCl_2_, 1.2 mM MgSO_4_, and 25 mM NaHCO_3_ with 4-(2-hydroxyethyl)-1-piperazineethanesulfonic acid (HEPES) plus 0.5% bovine serum albumin containing 0.5 μM fura-2/AM (Invitrogen, Grand Island, NY, USA), 8 μM pluronic acid F127 (Sigma-Aldrich, St. Louis, MO, USA) and 250 μM sulfinpyrazone (Sigma-Aldrich, St. Louis, MO, USA) for 1h. Before Ca^2+^ measurements, cells were washed with KRB-HEPES containing sulfinpyrazone. Ca^2+^ measurements were conducted using a ratio imaging system (In Cyt Im2; Intracellular Imaging, Cincinnati, OH, USA) using excitation wavelengths of 340 and 380 nm and an emission wavelength of 505 nm.

### Western blot:

Western blotting (WB) was conducted as previously described^46^. Total protein was separated by sodium dodecyl sulfate-polyacrylamide gel electrophoresis (SDS-PAGE) using Mini-PROTEAN^®^ 4–20% Precast Gels (Biorad, Hercules, CA, USA) and transferred to 0.45 μm pore-size nitrocellulose membrane. The membranes were blocked with 5% non-fat milk (Biorad, Hercules, CA, USA) at room temperature for 1 h and then incubated overnight at 4°C with the primary antibodies listed in Table 1. After being washed with TBS-Tween 20 (TBST) buffer, the membranes were incubated with horseradish peroxidase (HRP)-conjugated secondary antibody (BioRad, Hercules, CA, USA) Goat Anti-Rabbit IgG (H + L)-HRP (1:2000) or Goat Anti-Mouse IgG (H + L)-HRP (1:2000) for 1 h at room temperature. Signals were developed with enhanced chemiluminescence with a Clarity Western ECL Substrate (Biorad, Hercules, CA, USA) and detected with an iBright 1500 gel documentation system (Thermo Fisher Scientific, Waltham, MA, USA). Densitometry analysis was performed using ImageJ software (NIH, Bethesda, MD, USA). The whole uncropped and unprocessed membrane images overlayed with protein standard ladder are presented in Supplemental Figs. 2 and 6.

#### RT-qPCR

Total RNA was extracted using Quick RNA miniprep kit (Zymo Research, Santa Cruze, CA); the concentration and purity of the RNA were analyzed using a NanoDrop One spectrophotometer (Thermo Fisher Scientific, Waltham, MA, USA). cDNA was synthesized from 500 ng of total RNA using the PrimeScript RT Master Mix (RR036A, Takara, San Jose, CA, USA) on an Applied Biosystems 2720 thermal cycler (Life Technologies, Waltham, MA, USA). Quantitative PCR was performed on a CFX384 Touch Real-Time PCR Detection System (BioRad, Hercules, CA) using iTaq Universal SYBR Green Supermix (BioRad, Hercules, CA) and mouse-specific primers (Table 2). The PCR cycling conditions were initial denaturation at 95°C for 5 minutes, followed by 40 cycles of 95°C for 30 seconds, 60°C for 30 seconds (annealing), and 72°C for 30 seconds (extension). Each sample was run in duplicates. Fluorescence was recorded at the end of each cycle. Gene expression was normalized to β-actin, and fold changes were calculated using the 2^−ΔΔCT method.

#### Bulk RNA sequencing

Total RNA from trigeminal ganglia tissue was extracted using Quick RNA miniprep kit (Zymo Research, Santa Cruze, CA). Messenger RNA was purified, and quality was evaluated using an Agilent Bioanalyzer, and only samples with RNA Integrity Number (RIN) > 7 was used for RNA sequencing. RNA-seq libraries were prepared by Novogene (Sacramento, CA, USA) using poly(A) enrichment and sequenced on an Illumina NovaSeqX Plus platform to generate 150 bp paired-end reads at a depth of 30 million reads per sample. FastQC was used for quality control, Salmon 1.10.2 was used for alignment^47^, and DESeq2 was used for count normalization and statistical comparisons. Benjamini-Hochberg method was used to adjust the *p*-value (adj.p), and genes with adj.p < 0.05 were used for pathway analysis using The Gene Ontology (GO) knowledgebase and KEGG database. The gene network figure is made by STRING database^48^.

#### Statistical analysis

Investigators were blinded to treatment during the outcome assessment and statistical analysis. The data are presented as the fold-increase above basal or average ± standard error. The student’s *t*-test was used in 2 group comparisons, and One-way ANOVA with Tukey correction was used in multiple group comparisons. The *p*-value of less than 0.05 was considered statistically significant.

## Supplementary Material

This is a list of supplementary files associated with this preprint. Click to download.

- supplementarymaterials.pdf

## Figures and Tables

**Figure 6:**
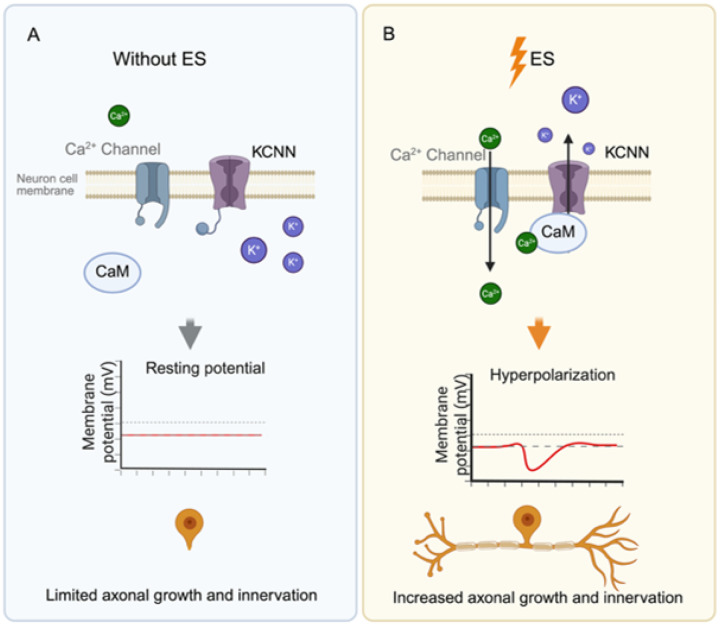
Schematics of the molecular action by ES in peripheral sensory neurons. (A) KCNN channel at resting state without ES; (B) KCNN activation upon ES application.

**Table 1:** Primary antibodies and dilutions used in the study

Target protein	Produced by	Product no	IHC dilution	WB dilution
β-III-tubulin	R&D, Minneapolis, MN, USA	NL1195R	1:30	-
GAP43	Thermo Fisher Scientific, Waltham, MA, USA	33–5000	1:100	1:2000
GAP43 Conjugated	Novus Biologicals, Centennial, CO, USA	NB300–143AF488	1:100	-
Akt	Cell Signaling, Danvers, MA, USA	9272	-	1:1000
p-Akt	Cell Signaling, Danvers, MA, USA	9271	-	1:1000
β-actin	Thermo Fisher Scientific, Waltham, MA, USA	MA5–15739	-	1:4000

**Table 2:** RT-PCR Primer list used in the study

Target gene	Primer Sequence	Source
*cacna1*	F: TCA GCA TCG TGG AAT GGA AAC	IDT (Coralville, IA, USA)
	R: GTT CAG AGT GTT GTT GTC ATC CT	
*kcnn1*	F: TTTAAAAGCGTAAACGGCTCA	IDT (Coralville, IA, USA)
	R:CAGAGCAAAAGAGCAGAGTGA	
*kcnn2*	F: TCCGTCGTAGGAGGAGGTG	IDT (Coralville, IA, USA)
	R: AATTGTTGTGCTCCGGCTTAG	
*kcnn3*	F: GGTCATTGAGATTTAGCTGGCTG	IDT (Coralville, IA, USA)
	R: CTGTTGCACTCTTCTCCCACG	
*β-actin*	F: CATTGCTGACAGGATGCAGAAGG	IDT (Coralville, IA, USA)
	R: TGCTGGAAGGTGGACAGTGAGG	

## Data Availability

All data generated or analyzed during this study are included in this published article, and its supplementary information files. Raw sequencing data are available following the anonymized link for the duration of the peer-review process; The data will switch to public upon acceptance. https://dataverse.harvard.edu/previewurl.xhtml?token=70846dd9-7a99-4f28-a65e-ddd6df6376df
